# Targeted Metabolic and Transcriptomic Analysis of *Pinus yunnanensis* var. *pygmaea* with Loss of Apical Dominance

**DOI:** 10.3390/cimb44110371

**Published:** 2022-11-03

**Authors:** Feng Xiao, Yang Zhao, Xiurong Wang, Yao Yang

**Affiliations:** 1 Institute for Forest Resources and Environment of Guizhou/Key Laboratory of Forest Cultivation in Plateau Mountain of Guizhou Province/College of Forestry, Guizhou University, Guiyang 550025, China; 2 Key Laboratory of Plant Resource Conservation and Germplasm Innovation in Mountainous Region (Ministry of Education), Guizhou University, Guiyang 550025, China

**Keywords:** *Pinus yunnanensis* var. *pygmaea*, loss of apical dominance, endogenous phytohormone, comparative transcriptomes

## Abstract

*Pinus yunnanensis* var. *pygmaea* demonstrates obvious loss of apical dominance, inconspicuous main trunk, which can be used as an ideal material for dwarfing rootstocks. In order to find out the reasons for the lack of apical dominance of *P. pygmaea*, endogenous phytohormone content determination by liquid chromatography–tandem mass spectrometry (LC–MS/MS) and comparative transcriptomes were performed on the shoot apical meristem and root apical meristem of three pine species (*P. massoniana*, *P. pygmaea*, and *P. elliottii*). The results showed that the lack of CK and the massive accumulation of ABA and GA-related hormones may be the reasons for the loss of shoot apical dominance and the formation of multi-branching, the abnormal synthesis of diterpenoid biosynthesis may lead to the influence of GA-related synthesis, and the high expression of *GA 2-oxidase* (*GA2ox*) gene may be the cause of dwarfing. Weighted correlation network analysis (WGCNA) screened some modules that were highly expressed in the shoot apical meristem of *P. pygmaea*. These findings provided valuable information for identifying the network regulation of shoot apical dominance loss in *P. pygmaea* and enhanced the understanding of the molecular mechanism of shoot apical dominance growth differences among *Pinus* species.

## 1. Introduction

Apical dominance is the control exerted by the apical portions of the shoot over the outgrowth of the lateral buds [1], which can be inhibited physically by the manual removal of apical buds or chemically by the application of plant growth regulators. After apical removal, sugar is rapidly redistributed throughout the plant and accumulated in axillary buds [2]. *BRANCHED 1* (*BRC1*) is considered to be an important hub of different signals controlling the ability of a bud to grow out in many species [3]. Artificially increasing sucrose levels in plants represses the expression of maintaining bud dormancy *BRC1* gene, and results in rapid bud release [4]. Sugar and abscisic acid (ABA) are responsible for initial release of a bud, while auxin, strigolactone (SL) and cytokinin (CK) determine sustained outgrowth [5]. The second messenger model proposes that CKs and SLs act downstream of auxin to control shoot branching [6]. Due to factors such as high heterozygosity, complex origin, long growth cycle, and difficulty in genetic analysis, the trait of apical dominance has an important utilization value in forestry production, but the research on its mechanism is far behind other model plants.

*Pinus massoniana* Lamb. (Fam.: *Pinus*; Gen.: *Pinus*) is a widely distributed tree species in China and plays an important role in ecological environment construction and sustainable forestry production. *P. elliottii* trees grow to 30 m tall, with trunks up to 0.8 m diameter at breast height in native range. *P. yunnanensis* var. *pygmaea* demonstrates obvious loss of apical dominance, inconspicuous main trunk; the base is multi-branched, clump-like, ranging from 40–50 cm to 1–2 m in height; leaves are upward and short; seed cones clustered [7,8]; and the adult tree is obviously dwarfed and maintains the dwarf nature. Natural apical dominance deletion mutants are beneficial to deepen the understanding of plant apical material synthesis and transport. Compared with *P. elliottii*, *P. massoniana* grows slower in the early stage (10–15 years) and faster in the later stage (>15 years). The growth rate of plant height in the early stage of the three species is *P. elliotti* > *P. massoniana* > *P. pygmaea.* After the truncation of *P. massoniana*, the dormancy of the axillary buds was released, the meristems were activated, the peripheral meristems continued to differentiate to form new scale leaves [9], the contents of indole-3-acetic acid (IAA), zeatin (ZT) and SL in lateral buds significantly increased after removal of apical dominance in *P. massoniana*, while ABA decreased [10]. In the construction of seed orchards, the main morphological characteristics of an ideal *P. massoniana* mother tree are: wide crown width, long branch length, large branch angle, wide crown, oval or spherical shape, which can only be dwarfed by cutting the trunk [11], and the labor consumption is large. Dwarfing rootstocks can enable high-density planting; the dwarf mutant of *P. pygmaea* is the first choice for grafting as a dwarfing rootstock. However, it is not known why the lack of apical dominance occurs in *P. pygmaea*.

The transcriptome sequencing (RNA-seq) can reveal the molecular components of tissues and cells, and understanding development [12]. Although chromosome-level assembly of *P. tabulaeformis* has been published, which proved the limits due to the existence of numerous huge intergenic regions and long introns with high transposable element, RNA-seq is more appropriate to reveal various molecular mechanisms in conifer species [13,14]. Plant hormones have been confirmed to be closely related to plant growth and development [15]. Liquid chromatography–tandem mass spectrometry (LC–MS/MS) has been widely used for hormone determination in the past few years [16]. In this study, endogenous phytohormone content determination by LC–MS/MS and RNA-seq were performed on the shoot apical meristem (SAM) and root apical meristem (RAM) of three pine species (*P. massoniana*, *P. pygmaea* and *P. elliottii*), in order to find out the reasons for the lack of apical dominance of *P. pygmaea*, providing theoretical guidance for the construction of seed orchards and the production and application of pine trees.

## 2. Materials and Methods

### 2.1. Acquisition of Test Materials

After early investigation and collection of provenance, the collected half-sib progeny of *P. massoniana*, *P. pygmaea* and *P. elliotti* seeds were planted at the same time in a nursery greenhouse after germination treatment. The soil type in the pot was humus:yellow loam soil (1:3). The SAM (A) of annual *P. massoniana*, *P. pygmaea* and *P. elliotti*, and their respective RAM tissues (R), were collected at Guizhou University in September 2021, then washed, immediately frozen in liquid nitrogen and stored at −80 °C; three samples were taken as biological replicates.

### 2.2. Plant Hormone Determination by LC-MS/MS

Samples (50 mg fresh weight) were ground to a powder (30 Hz, 1 min) with a grinder (MM 400, Retsch) and extracted with methanol/water/formic acid (15:4:1, *v*/*v*/*v*). The extracts were concentrated and reconstituted with 80% methanol-water solution, then filtrated (PTFE, 0.22 μm; Anpel) before LC-MS/MS analysis. Endogenous hormone contents were detected by MetWare (http://www.metware.cn/ (accessed on 1 November 2021)) based on the AB Sciex QTRAP 6500 LC-MS/MS platform. A one-way analysis of variance (ANOVA) in combination with Duncan’s multiple range test with a significance of differences of *p* < 0.05 was conducted by R v4.1.3 (https://www.r-project.org/ (accessed on 1 May 2022)). 

### 2.3. RNA Extraction and Transcriptomic Library Construction

Total RNA was isolated using Trizol kit (Invitrogen, Waltham, MA, USA). RNA samples were analyzed for concentration and quality using NanoDrop 2000 (Thermo Fisher Scientific, Waltham, MA, USA). The 28S/18S ratio and RIN values was determined using an Agilent 2100 system (Agilent Technologies, Santa Clara, CA, USA). RNA integrity was assessed by agarose gel electrophoresis. Total RNA samples with RIN ≥ 8.0 and 2.0 < OD260/280 < 2.2 were used for constructing the cDNA libraries. The mRNA was enriched with Oligo (dT) magnetic beads, and the mRNA was added with fragmentation buffer and cut into short fragments. Using the interrupted mRNA as templates, cDNA was reverse transcribed using six-base random primers. The double-stranded cDNA samples were purified, end-repaired, added with poly (A) tails and then ligated to the sequencing adapters to create cDNA libraries. After the libraries passed quality test, they were sequenced by the Illumina HiSeq X Ten with 150 bp paired-end. The raw data were stored in the NCBI/SRA database (BioProject accession No.: PRJNA863936).

### 2.4. Comparative Transcriptomic Analysis

Quality control on raw data was conducted using fastp v0.12.4 tool [17], and Pacific Biosciences (Pacbio) single molecule real time (SMRT) transcriptome of *P. massoniana* was used as a reference [18]. Bowtie2 v2.4.1 [19] was used to align the sequenced transcriptomic data. The fragments per kilobases of the transcript per million fragments mapped (FPKM) values was used to indicate gene expression levels by RSEM (https://github.com/deweylab/RSEM (accessed on 1 May 2022)) [20]. The differentially expressed genes (DEGs) were identified based on the read count using DESeq2 v1.34.0 [21]; DEGs screening thresholds were set as *p*-value < 0.05 & |foldchange| > 2. DEGs clusters and visualize genes with similar expression patterns were performed by the Mfuzz v2.54.0 R package [22], divided the DEGs into 8 clusters; the minimum score threshold was set to 0.25. Gene ontology (GO) and Kyoto Encyclopedia of Genes and Genomes (KEGG) enrichment analysis were performed by the clusterProfiler v4.2.2 R package [23]. Weighted gene co-expression network analysis was performed by WGCNA v1.71 R package [24], constructing a co-expression network for all genes and all samples. The top 5000 genes were screened by median absolute deviation (MAD) for further analysis, parameters were set up as power = 13, minModuleSize = 30, MEDissThres = 0.25. To identify significant modules related to traits, the association of gene significance (GS) and module membership (MM) were evaluated.

## 3. Results

### 3.1. Differences in Endogenous Phytohormone Content in Different Annual Pine Seedlings

**Figure 1:**
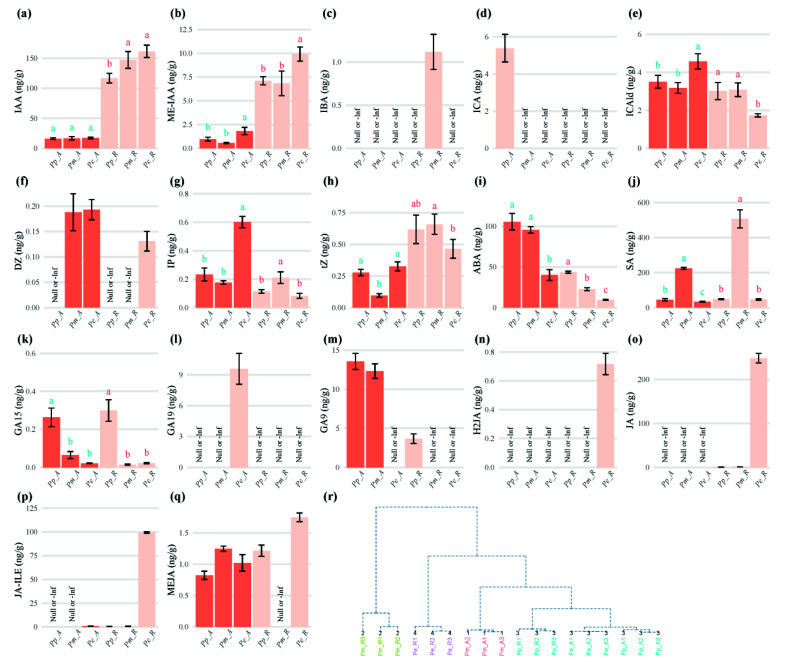
Determination of endogenous phytohormone contents in three pine species. In (**a**–**q**), they were histogram of IAA, ME-IAA, IBA, ICA, ICAld, DZ, IP, tz, ABA, SA, GA_15_, GA_19_, GA_9_, H_2_JA, JA, JA-lie, MeJA respectively. (**r**) The cluster analysis of endogenous phytohormone contents. Note: In (**a**–**q**), error bars represented means ± SD (n = 3). Multiple comparative analysis was performed using LSD.test analysis, different label with colors represented the results of one-way ANOVA of different parts, the group with no signal was marked as “Null or -Inf”. *Pm*, *Pinus massoniana*; *Pp*, *Pinus yunnanensis* var. *pygmaea*; *Pe*, *Pinus elliottii*; A, shoot apical meristem; R, root apical meristem; In (**r**), the method adopted was hclust cluster, the number of clusters was 4.

By measuring the endogenous phytohormone contents in the SAM and RAM of three pine seedlings, the IAA content of the root tips of the three pines was, from large to small: *Pe_R > Pp_R > Pm_R*; the distribution ranged from 117 to 161.7 ng/g, while there was no difference in the IAA content of the SAM of the three pine species (Figure 1a). The content distribution of methyl indole-3-acetate (ME-IAA) and IAA were similar, and the RAM content was significantly larger than the SAM (Figure 1b). 3-indolebutyric acid (IBA) was only detected in the roots of *P. massoniana*, the content was 1.12 ± 0.2 ng/g (Figure 1c); Indole-3-carboxylic acid (ICA) was only detected in the SAM of *P. pygmaea*, at 5.39 ± 0.74 ng/g (Figure 1d), while indole-3-carboxaldehyde (ICAld) was distributed in two tissue sites of the three pines (Figure 1e); Dihydrozeatin (DZ) was not detected at the SAM and RAM of the *P. pygmaea* (Figure 1f). The n6-isopentenyladenine (IP) content at SAM (0.6 ± 0.04 ng/g) of *P. elliotti* was significantly larger than that of the RAM (0.08 ± 0.017 ng/g) (Figure 1g). The trans-zeatin (tz) content in the RAMs was significantly higher than those in their SAMs (Figure 1h). The content of ABA was the highest in the SAM of *P. pygmaea* at 105.6 ± 10.17 ng/g (Figure 1i). The salicylic acid (SA) content in the *P. massoniana* was significantly higher than that of *P. elliotti* and *P. pygmaea* (Figure 1j). The content of GA_15_ in *P. pygmaea* was significantly higher than that of *P. massoniana* and *P. elliotti* (Figure 1k). GA_19_ was only detected at the SAM of *P. elliotti* (Figure 1l). The content of GA_9_ in the SAM was significantly higher than that in the RAM (Figure 1m). Dihydrojasmonic acid (H_2_JA) was detected only at the root tip of *P. elliotti* (Figure 1n). Jasmonic acid (JA), JA-lie (jasmonoyl-l-isoleucine), meJA (methyl jasmonate) were highest in the RAM of *P. elliotti* (Figure 1o–q). Cluster analysis showed that the RAM of *P. pygmaea* were clustered together with the SAM of *P. elliotti* (Figure 1r).

### 3.2. Characteristic Analysis of DEGs in Different Pine Species

**Figure 2:**
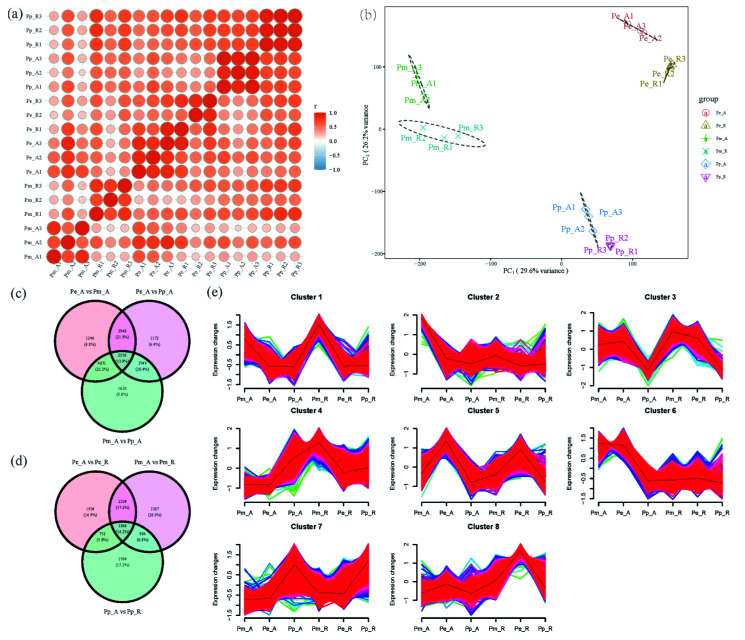
Correlation analysis of transcriptome samples and trend analysis of DEGs. (**a**) Sample correlation heatmap; (**b**) sample PCA analysis; (**c**) Venn diagram of DEGs; (**d**) Venn diagram of DEGs; (**e**) Trend analysis of DEGs by Mfuzz. Note: In (e), the default color palette was used, the black line represented the cluster centre.

After quality control of transcriptome raw data, based on the comparison of the Pacbio SMRT transcriptome of *P. massoniana*, the Pearson correlation analysis was performed on the mRNA expression levels of all samples; there was a high correlation between samples in the same group (Figure 2a). The contribution rate of PC_1_ was 29.6%, the contribution rate of PC_2_ was 26.2%, and the cumulative contribution rate was high (Figure 2b). According to the screening criteria of DEGs, there were down-regulated 4655 genes and up-regulated 7335 genes between Pm_A and Pp_A. There were down-regulated 6512 genes and up-regulated 5907 genes between Pe_A and Pp_A group. There were down-regulated 8088 genes and up-regulated 3727 genes between Pe_A and Pm_A group. A total of 1846 DEGs (14.2%) were compared between the SAM and RAM of various species (Figure 2d). There were 6299 union DEGs between the Pm_A vs. Pp_A and Pe_A vs. Pp_A and the trend analysis of these shared DEGs were divided into 8 trends (Figure 2e). Among them, genes in cluster3 showed relatively lower expression at Pp_A, genes in cluster7 showed relatively higher expressed at Pp_A. GO enrichment analysis of gene list in cluster3 showed that, ligase activity (GO: 0016874), phosphopyruvate hydratase activity (GO: 0004634) molecular function (MF) terms were enriched, purine nucleoside triphosphate biosynthetic process (GO:0009145), purine ribonucleoside monophosphate biosynthetic process (GO:0009168) and other biological process (BP) terms were enriched; mitochondrial envelope (GO:0005740) cellular component (CC) term was enriched; cluster7, contained *gibberellin regulated protein* (transcript_41812), *PIN 2* (transcript_5515), *ABC transporter* (transcript_10268, transcript_31907, transcript_68, transcript_7697) and other genes. GO enrichment analysis of gene list in cluster7 showed that MF terms such as oxidoreductase activity, oxidizing metal ions (GO:0016722) and calcium ion binding (GO:0005509) were enriched.

### 3.3. GO and KEGG Enrichment Analysis of DEGs

**Figure 3:**
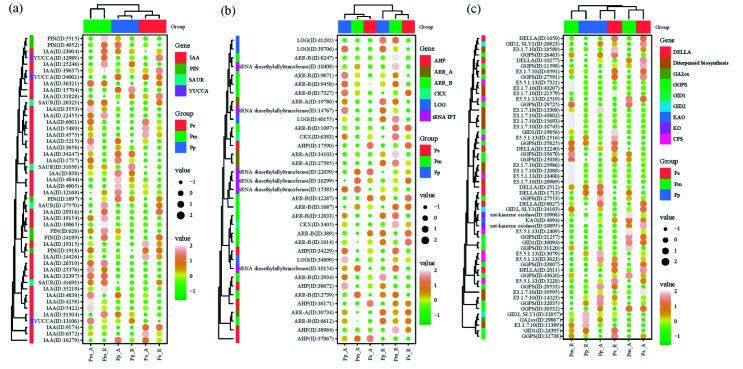
The DEGs involved in the synthesis and response of IAA, CK, GA. (**a**) The DEGs involved in the synthesis and response of IAA; (**b**) the DEGs involved in the synthesis and response of CK; (**c**) the DEGs involved in the synthesis and response of GA. Note: *SAUR*, small auxin upregulated RNA; *ARR_A*, two-component response regulator ARR-A family; *ARR_B*, two-component response regulator ARR-B family; *AHP*, histidine-containing phosphotransfer peotein; *CKX*, cytokinin dehydrogenase; LOG, LONELY GUY; tRNA IPT, tRNA dimethylallyltransferase; *GA2ox*, GA 2-oxidase; *GGPS*, geranylgeranyl pyrophosphate synthase; *KAO*, ent-kaurenoic acid oxidase; *KO*, ent-kaurene oxidase; CPS, ent-copalyl diphosphate synthase.

GO enrichment analysis was performed on the DEGs of Pm_A vs. Pp_A group. BP terms, such as photosynthesis (GO:0015979), chlorophyll metabolic process (GO:0015994) were enriched, CC terms such as thylakoid lumen (GO:0031977), nucleoid (GO:0009295), plastid large ribosomal subunit (GO:0000311), were enriched; protein disulfide oxidoreductase activity (GO: 0015035), glucose binding (GO: 0005536), monosaccharide binding (GO: 0048029), glucosyltransferase activity (GO: 0046527) and other MF terms were enriched. Carbon fixation in photosynthetic organisms (map00710), diterpenoid biosynthesis (map00904), phenylpropanoid biosynthesis (map00940) and other KEGG pathways were enriched. In the diterpenoid biosynthesis pathway, relative to *P. massoniana*, six *(13E)-labda-7*, *13-dien-15-ol synthase genes* (*LDS*, transcript_11389, transcript_13308, transcript_14223, transcript_18588, transcript_20969), *diterpene synthase* (transcript_14223) and *delta-selinene synthase* (transcript_13308), *longifolene synthase* (transcript_20969) were up-regulated in *P. pygmaea* (Figure 3). In addition, *GA 2-oxidase* (*GA2ox*, transcript_29867) was relatively highly expressed in the SAM and RAM of *P. pygmaea*.

GO enrichment analysis was performed on the DEGs at the Pe_A vs. Pp_A group, response to karrikin (GO: 0080167), plastid organization (GO: 0009657). BP terms were enriched; MF terms such as glucose binding (GO:0005536) and monosaccharide binding (GO:0048029) were enriched; pentose and glucuronate interconversions (map00040) KEGG pathway was enriched. The KEGG enrichment analysis of the DEGs at the Pm_A vs. Pe_A showed that the citrate cycle (TCA cycle) (map00020) was significantly enriched. 

### 3.4. WGCNA Analysis

**Figure 4:**
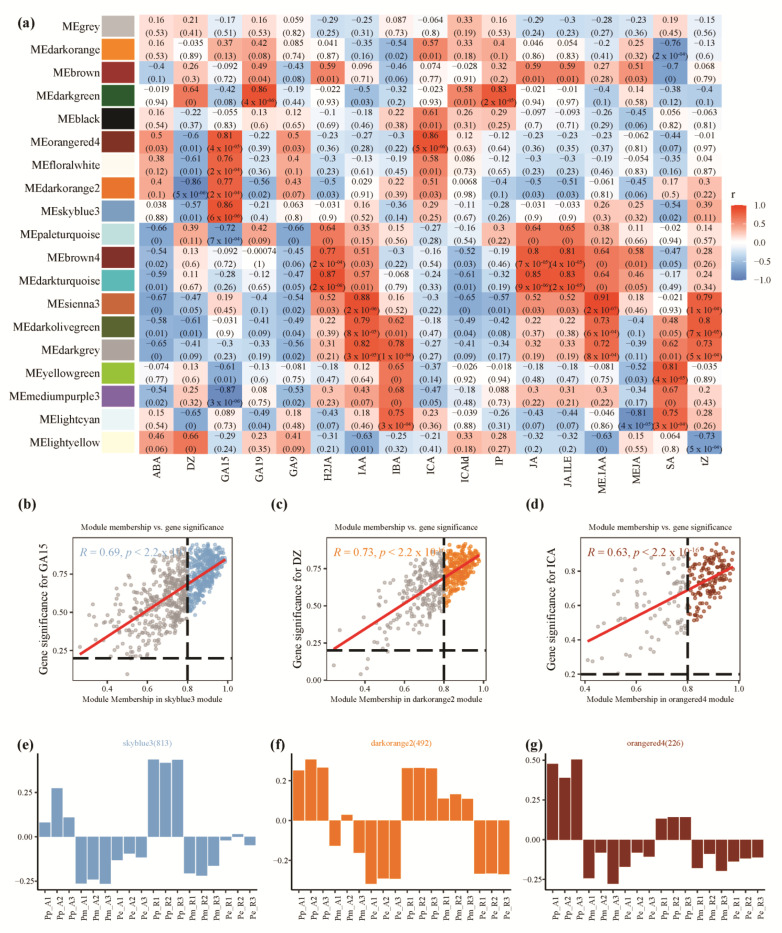
Weighted gene co-expression network analysis of the genes. (**a**) Correlated heatmap of the adjacency of modules; (**b**) a scatterplot of GS for GA_15_ vs. MM in the skyblue3 module; (**c**) a scatterplot of GS for DZ vs. MM in the darkorange2 module; (**d**) a scatterplot of GS for ICA vs. MM in the orangered4 module; (**e**) the eigengene expression of skyblue3 module; (**f**) the eigengene expression of darkorange2 module; (**g**) the eigengene expression of orangered4 module. Note: In (**a**), each row represented a module, the color and number of each cell represented the correlation coefficient between modules and traits, the top number in the cell represented the correlation coefficient and the bottom number represented the *p*-value. In (**b**–**d**), GS was calculated as the absolute value of the correlation between expression profile and each trait, MM was defined as the correlation of expression profile and each module eigengene and red represented the linear regression line.

WGCNA analysis showed that the co-expression network could be divided into 19 modules. The skyblue3 module had a highly positive correlation with GA_15_ (correlation coefficient (*r*) = 0.86, *p*-value (*p*) = 6 × 10^−6^) (Figure 4a), which was enriched with aerenchyma formation (GO:0010618), regulation of hydrogen peroxide metabolic process (GO:0010310), response to reactive oxygen species (GO:0000302), response to nutrient (GO:0007584) and other BP terms. The module membership in the skyblue3 module and the gene significance have a high correlation (*r* = 0.69, *p* < 2.2 × 10^−16^), suggesting that the module is suitable for identifying the hub genes associated with the staging of GA_15_ (Figure 4b). The eigengene genes in the skyblue3 module were mainly highly expressed in Pp_A and Pp_R tissues (Figure 4e).

The darkorange2 module had a highly negative correlation with DZ (*r* = −0.86, *p* = 5 × 10^−6^), and a highly positive correlation with DZ (*r* = 0.64, *p* = 0.004). The module membership in the orangered4 module and the gene significance have a high positive correlation (*r* = 0.73, *p* < 2.2 × 10^−16^) (Figure 4c). The eigengene genes in the darkorange2 module were mainly highly expressed in Pp_A, Pp_R and Pm_R (Figure 4f).

The orangered4 module was significantly positively correlated with multiple traits, significantly positively correlated with GA_15_ (*r* = 0.81, *p* = 4 × 10^−5^) and ICA (*r* = 0.86, *p* = 5 × 10^−6^), the genes were relatively highly expressed at the SAM of *P. pygmaea* in the orangered4 module (Figure 4c). GO enrichment of the genes in the orangered4 module showed that mitochondrial mRNA modification (GO:0080156), terpene biosynthetic process (GO:0046246), terpene metabolic process (GO:0042214) and other BP terms were enriched. The module membership in the orangered4 module and the gene significance have a high positive correlation (*r* = 0.63, *p* < 2.2 × 10^−16^) (Figure 4d). The eigengene expression in the orangered4 module were mainly highly expressed in Pp_A (Figure 4g). The sienna3 module had a highly positive correlation with IAA (*r* = 0.88, *p* = 2 × 10^−6^), MeIAA (*r* = 0.91, *p* = 2 × 10^−7^), the sienna3 module had highly negative correlations with ABA (*r* = −0.67, *p* = 0.002). 

## 4. Discussion

*P. pygmaea* is a variant of *P. yunnanensis* that demonstrates obvious loss of apical dominance; the base is multi-branched. The changes in branch growth traits of *P. pygmaea* population before and after a 10-year period were compared. The ratio of height and length increased by 36.3% [25]; the twisted and low form indicates adaptation to higher altitudes and worse ecological conditions [26]. *P. pygmaea* is the first choice for grafting as a dwarfing rootstock, which is an excellent material for studying apical dominance of genus *Pinus*. Plant height growth reflects the strength of apical dominance. In recent years, research on the formation of apical dominance has mainly focused on the synthesis, transport, signal transduction and metabolism of plant hormones. For instance, GA, BR, auxin and SLs control plant height through regulating cell elongation and cell [27]. GA and IAA have significant effects on banana dwarfing [28]. In other dwarf pine species, endogenous phytohormones are associated with dwarf formation. The loss of apical dominance in the *P. sylvestris* var. *mongolica* was accompanied by a significant decrease in IAA and CK content compared to wild type [29]. The characteristics of dwarfed variant *P. bungeana*, short plants and numerous lateral branches, may be closely related to the significant increase in ZT, the decrease in IAA/ABA and the increase in ZT/IAA [30]. The interaction between CK and IAA is required for the regulation of the SAM and the RAM [31]. The *WUSCHEL* (*WUS*) gene, which is specifically expressed in the organizing center (QC), is responsible for SAM formation, whereas the QC-expressed *WUSCHEL-RELATED HOMEOBOX 5* (*WOX5*) gene plays a key role in RAM formation, ectopic expression of *WOX5* disrupted shoot development by repressing shoot-related genes, such as *YABBY1* [32]. However, *WUS* homeobox genes were not specifically expressed in the three pine species. There was no difference in the IAA content in the SAM of the three pine species (Figure 1a), but the IAA content of the RAM of *P. pygmaea* was significantly less than that in the other two species. The apparent decreased in IAA hormone levels in the roots of *P. pygmaea* may be due to the distribution of apical IAA hormones to multi-branched. 

The major shoot signal produced in the apical bud and young leaves is auxin, while the basic root tip signals produced in the root cap are CKs [33]. The phenotype of dwarf phenotype pears could be primarily attributed to deficiencies in cell division [34]. *ATP/ADP isopentenyltransferase* (*IPT*) is a key CK biosynthesis genes; after decapitation, the expression levels of *PpIPT1*, *PpIPT3* and *PpIPT5a* in nodal stems sharply increased in peach [35]. Deletion variant is the core site for promoter activity, which is located at 1172 bp upstream of ATG in the *IPT5b* gene between apple *M9* rootstock (dwarfing) and *Robusta* rootstock (vigorous), low *IPT5b* expression with high level methylations in promoter region, leading to poor root tz biosynthesis in the M9 rootstock, which may induce dwarfing [36,37]. Plant growth inhibitors exogenously applied to plants can be used in dwarf cultivation [38]. Foliar spraying with 400 mg·L^−1^ 6-Benzyladenine (6-BA) inhibited the plant height of adult tea tree by 22.0% [39]. The content of tz in the RAMs of the three pine species was greater than that in the corresponding SAMs (Figure 1h). Excessive accumulation of ABA can lead to dwarfing of mutant ‘601T’ pears [40]. *Arabidopsis* β-glucosidase (*AtBG1*) can hydrolyze glucose-conjugated, biologically inactive ABA to produce active ABA, the transgenic bent grass plants overexpressing *AtBG1* had a dwarf phenotype with reduced growth rates [41]. By measuring the hormone content of the SAM of three pine species, DZ was not detected at the SAM and RAM of the *P. pygmaea* (Figure 1f). The content of ABA was the highest in the SAM of *P. pygmaea* at 105.6 ± 10.17 ng/g (Figure 1i). The content of GA_15_ in *P. pygmaea* was significantly higher than that of *P. massoniana* and *P. elliotti* (Figure 1k). This indicated that the lack of CK and the massive accumulation of ABA and GA-related hormones may be the reasons for the loss of shoot apical dominance and the formation of multi-branching in *P. pygmaea*.

Differences in gene expression between mutant and normal types may be responsible for the altered traits. Genes associated with disease and stress responses are up-regulated in dwarf soybean compared to normal [42]. The dwarf phenotype of T51 of *seashore paspalum* is closely related to the abnormal synthesis of lignin and flavonoids in the phenylpropane pathway [43]. Diterpenoid biosynthesis (map00904), phenylpropanoid biosynthesis (map00940) and other KEGG pathways were enriched between the Pm_A and Pp_A group. In the Diterpenoid biosynthesis pathway, relative to *P. massoniana*, six *LDS* genes (transcript_11389, transcript_13308, transcript_14223, transcript_18588, transcript_20969), *diterpene synthase* (transcript_14223) and *delta-selinene synthase* (transcript_13308), *longifolene synthase* (transcript_20969) was up-regulated in *P. pygmaea* (Figure 3). *LDS* is the crucial enzyme of (13E)-Labda-7,13-dien-15-ol biosynthesis [44], *LDS* was identified one of the candidate genes for screening higher oleoresin yield of *P. massoniana* [45]. The high expression of LDS gene may be due to the multi-branching of *P. pygmaea*. 

The WGCNA analysis found that in several modules highly associated with GAs, the orangered4 module was significantly positively correlated with GA_15_ (*r* = 0.81, *p* = 4 × 10^−5^) and ICA (*r* = 0.86, *p* = 5 × 10^−6^) (Figure 4a); these genes were relatively highly expressed at the SAM of *P. pygmaea* (Figure 4g). GO enrichment of the genes in the orangered4 module showed that in mitochondrial mRNA modification terpene biosynthetic process, terpene metabolic process and other BP terms were enriched. Gibberellin regulated protein (transcript_41812) showed relatively higher expressed at Pp_A (Figure 2e). *GA2ox* (transcript_29867) was relatively highly expressed in the SAM and RAM of *P. pygmaea*. Differential expression of *GA2ox* is considered to be a determinant of plant height in various plants. *GA2ox* were only up-regulated in dwarf cultivar litchi samples, indicating GA might play an important role in regulating difference between vigorous and dwarf cultivars [46]. *GA2ox* for GA and tryptophan decarboxylase (*TDC*) and *YUCCA* for IAA were the most associated with plant height in banana [28]. In *Chimonanthus praecox*, transcriptome differential gene analysis showed that the elevated expression of the *CpGA2ox* and *CpGAI* gene in the signal transduction pathway might be the key mechanisms leading to dwarfing [47]. These indicated that the abnormal synthesis of diterpenoid biosynthesis may lead to the influence of GA-related synthesis, and the high expression of *GA2ox* gene may be the cause of dwarfing.

## 5. Conclusions

This study reported the endogenous phytohormone content determination by LC-MS/MS and comparative transcriptome analysis of three pine species (*P. massoniana*, *P. pygmaea* and *P. elliottii*). The determination of endogenous phytohormone content showed that the lack of CK and the massive accumulation of ABA and GA-related hormones may be the reasons for the loss of shoot apical dominance and the formation of multi-branching. Comparative transcriptome analysis showed that the abnormal synthesis of diterpenoid biosynthesis may lead to the influence of GA-related synthesis, and the high expression of *GA2ox* gene may be the cause of dwarfing in *P. pygmaea*. This study will provide information for further study of *Pinus* dwarf-related genes.

## Data Availability

The raw reads generated from Illumina sequencing have been deposited in the NCBI SRA database (accession BioProject: PRJNA863936).
